# Regulation and functional significance of CDC42 alternative splicing in ovarian cancer

**DOI:** 10.18632/oncotarget.4865

**Published:** 2015-08-22

**Authors:** Xiaolong He, Chengfu Yuan, Jilai Yang

**Affiliations:** ^1^ Department of Biopharmaceutical Sciences, College of Pharmacy-Rockford, The University of Illinois at Chicago, Rockford, IL 61107, USA; ^2^ Medical College of China Three Gorges University, Yichang, Hubei, 443002, People's Republic of China

**Keywords:** alternative splicing, PTBP1, CDC42, ovarian cancer

## Abstract

Our previous study found that splicing factor polypyrimidine tract-binding protein 1 (PTBP1) had a role in tumorigenesis but the underlying mechanism remained unclear. In this study, we observed that knockdown of PTBP1 inhibited filopodia formation. Subsequently, we found that PTBP1 regulated the alternative splicing of CDC42, a major regulator of filopodia formation. Two CDC42 variants, CDC42-v1 and CDC42-v2, can be generated through alternative splicing. Knockdown of PTBP1 increased the expression of CDC42-v2. Ectopic expression of individual variants showed that CDC42-v2 suppressed filopodia formation, opposite to the effect of CDC42-v1. Quantitative RT-PCR revealed that CDC42-v2 was expressed at lower levels in ovarian cancer cell lines and ovarian tumor tissues than in normal control cells and tissues. Further, CDC42-v2 was observed to have inhibitory effects on ovarian tumor cell growth, colony formation in soft agar and invasiveness. In contrast, these inhibitory effects were not found with CDC42-v1. Taken together, above results suggest that the role of PTBP1 in tumorigenesis may be partly mediated by its regulation of CDC42 alternative splicing and CDC42-v2 might function as a tumor suppressor.

## INTRODUCTION

Alternative splicing is a major mechanism for the expansion of proteomic complexity from the limited number of genes, playing critical roles in normal development and physiology [1]. Misregulation of this process, which can be caused by alterations in the sequences of primary transcripts and/or regulatory proteins, has been associated with various human diseases, contributing to their initiation, progression and/or severity [2–4]. Unlike in other diseases, splicing changes in cancer cells are mostly ascribed to the alterations in *trans*-acting factors, such as serine/arginine-rich (SR) proteins and heterogeneous nuclear ribonucleoproteins (hnRNPs), rather than nucleotide mutations [5–7]. In our previous studies, we found that splicing factors polypyrimidine tract-binding protein 1 (PTBP1), a member of hnRNP family, and SRp20, a member of SR protein family, were overexpressed in human ovarian and breast cancer cells and their expression were correlated with malignant potential of ovarian tumors but not with the stages of invasive tumors. Knockdown of either PTBP1 or SRp20 caused substantial growth inhibition or apoptosis, indicating a requirement for their overexpression to maintain the transformation properties of tumor cells [8–10]. What remain unclear are the mechanisms mediating the roles of PTBP1 and SRp20 in human cancers.

CDC42 is a member of Rho GTPase family, involved in the regulation of a variety of cellular processes including cell polarity, cytoskeleton remodeling, migration, proliferation, trafficking and adhesion [11]. In the cell, CDC42 switches between two states: active GTP-bound state and inactive GDP-bound state [11]. Activation of CDC42 can be induced by diverse signals such as growth factors, cytokines and interactions between cells or between integrins and extracellular matrix [12]. Dysregulation of CDC42 activity has been associated with several disease states and developmental disorders, including cancer [13]. Accumulating evidence indicates that CDC42 has an important but complex role in cancer. Studies using constitutively active or dominant-negative CDC42 mutants showed that CDC42 was an oncoprotein, promoting cellular transformation and metastasis [14, 15]. However, gene knockout studies suggested that CDC42 might function as a tumor suppressor, because targeted ablation of CDC42 gene in the hepatocytes or blood stem/progenitor cells resulted in the development of hepatocellular carcinoma or myeloproliferative disease in mice [16, 17].

Human CDC42 gene is located on chromosome 1, from which three transcripts are derived via alternative splicing, which encode two distinct CDC42 variants, v1 and v2. The differences between two variants are in amino acid at position 163 and last 10 amino acids at c-terminus with v1 terminating in amino acids CVLL, a classical CaaX motif, while v2 terminating in CCIF. To date, the functional differences between two variants have been largely unknown because the great majority of studies on CDC42 have been conducted with CDC42-v1 or its mutants. Very recently, it has been found that v1 and v2 are differentially lipidated at c-terminus [18, 19]: CDC42-v1 is prenylated at Cys^188^ and undergoes end processing typical to CaaX motif, whereas CDC42-v2 has another type of modification besides the classical CaaX processing, that is, its Cys^189^ can be further palmitoylated after prenylation at Cys^188^. Dual lipidated CDC42-v2 displayed reduced binding activity with Rho GDP-dissociation inhibitor α (RhoGDIα) and was enriched in the plasma membrane compared to the prenylated-only CDC42 [18].

In an attempt to investigate the mechanisms behind the role of PTBP1 in tumorigenesis, we observed that PTBP1 knockdown inhibited filopodia formation and changed the splicing pattern of CDC42, a major regulator of filopodia formation. Subsequently, we found that CDC42-v1 and -v2 had different effects on filopodia formation and tumor cell behaviors. We also found that CDC42-v2 was downregulated in ovarian tumor cells. These results suggest that PTBP1 plays a role in tumorigenesis partly through its regulation of CDC42 splicing and CDC42-v2 might function as a tumor suppressor.

## RESULTS

### Knockdown of PTBP1 inhibits filopodia formation

**Figure 1:**
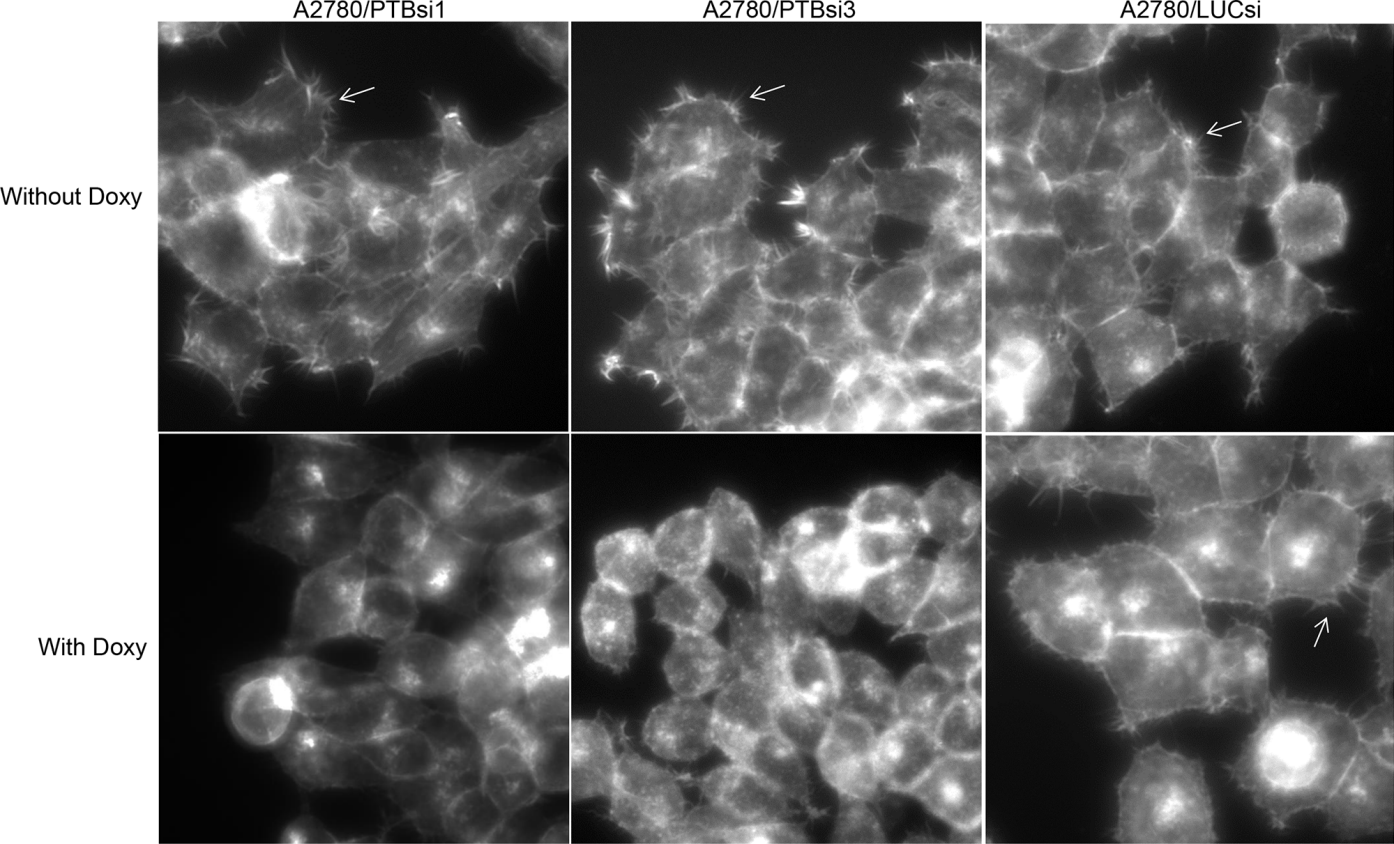
Knockdown of PTBP1 inhibits filopodia formation The arrows point to filopodia. Magnification: 400×.

Our previous study showed that PTBP1 played a role in the tumorigenesis of ovarian and breast cancer and regulated cellular metabolism by controlling the alternative splicing of pyruvate kinase [8–10]. In an attempt to identify other cellular processes that PTBP1 may regulate, we examined the effects of PTBP1 knockdown on actin cytoskeleton by staining F-actin using rhodamine phalloidin. Three sublines of ovarian cancer cell line A2780 were employed for this purpose. A2780/PTBP1si1 and A2780/PTBP1si3 are sublines expressing doxycycline (Doxy)-induced PTBP1 siRNA1 and siRNA 3, respectively, which suppressed the expression of PTBP1 by more than 75%, as demonstrated in our previous study [8]. The control subline, A2780/LUCsi, expresses a Doxy-induced luciferase siRNA. As shown in Figure 1, A2780/PTBP1si1 and A2780/PTBP1si3 cells grown in the absence of Doxy exhibit numerous microspikes, also called filopodia, on the cell surfaces, while the same subline cells grown in the presence of Doxy, i.e. with PTBP1 knockdown, have few or no such microspikes. In contrast, the control subline cells exhibit similar amount of filopodia when grown in the absence or presence of Doxy.

### Knockdown of PTBP1 changes alternative splicing of CDC42

**Figure 2:**
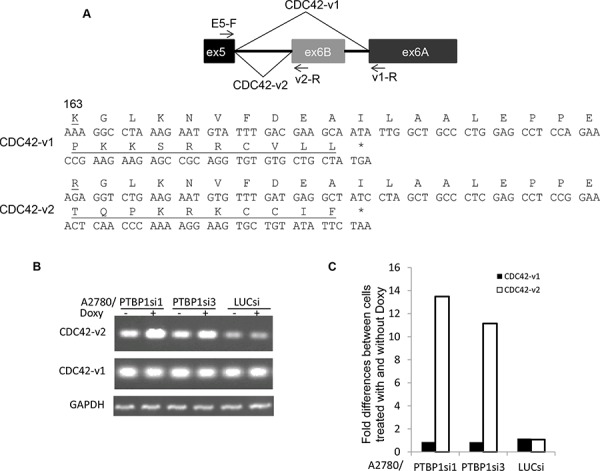
Knockdown of PTBP1 alters alternative splicing of CDC42 **A.** A diagram of alternative splicing of the terminal exons of human CDC42 (top) and the amino acid sequences encoded by exons 6A and 6B. The amino acids that are different between two are underlined. **B.** Representative regular RT-PCR showing the expression of CDC42 variants in cells treated with or without Doxy. **C.** Fold differences of CDC42 variants between cells treated with and without Doxy determined by qRT-PCR. Two separate experiments were performed and exhibited similar results and shown are the results of one experiment.

CDC42 is a major regulator of filopodia formation [20, 21] and has two isoforms (CDC42-v1 and CDC42-v2) that are generated through alternative splicing of the terminal exons. CDC42-v1 has exon 6A as the terminal exon while CDC42-v2 has exon 6B as the terminal exon (Figure 2A). Exon 6A and exon 6B each encode C-terminal 30 amino acids for CDC42-v1 and CDC42-v2, respectively, with the differences at amino acid 163 and the very C-terminal 10 amino acids (Figure 2A). We examined the expression of CDC42-v1 and CDC42-v2 by regular reverse transcription (RT)-PCR and quantitative RT-PCR (qPCR) using primer pairs with the common primer located on exon 5 (E5-F) and CDC42-v1-specific primer located on exon 6A (v1-R) or CDC42-v2 specific primer located on exon 6B (v2-R). As shown in Figure 2B, A2780/PTBP1si1 and A2780/PTBP1si3 cells grown in the presence of Doxy, i.e. with PTBP1 knockdown, expressed higher levels of CDC42-v2 and slightly lower levels of CDC42-v1 than the cells without PTBP1 knockdown. Quantitatively, CDC42-v2 was increased 11 to 13 folds while CDC42-v1 was reduced about 15% after PTBP1 knockdown (Figure 2C). The smaller change of CDC42-v1 compared to CDC42-v2 is because the former is the dominant isoform.

### PTBP1 represses the splicing of alternative terminal exon 6B through its interaction with upstream intron sequence

**Figure 3:**
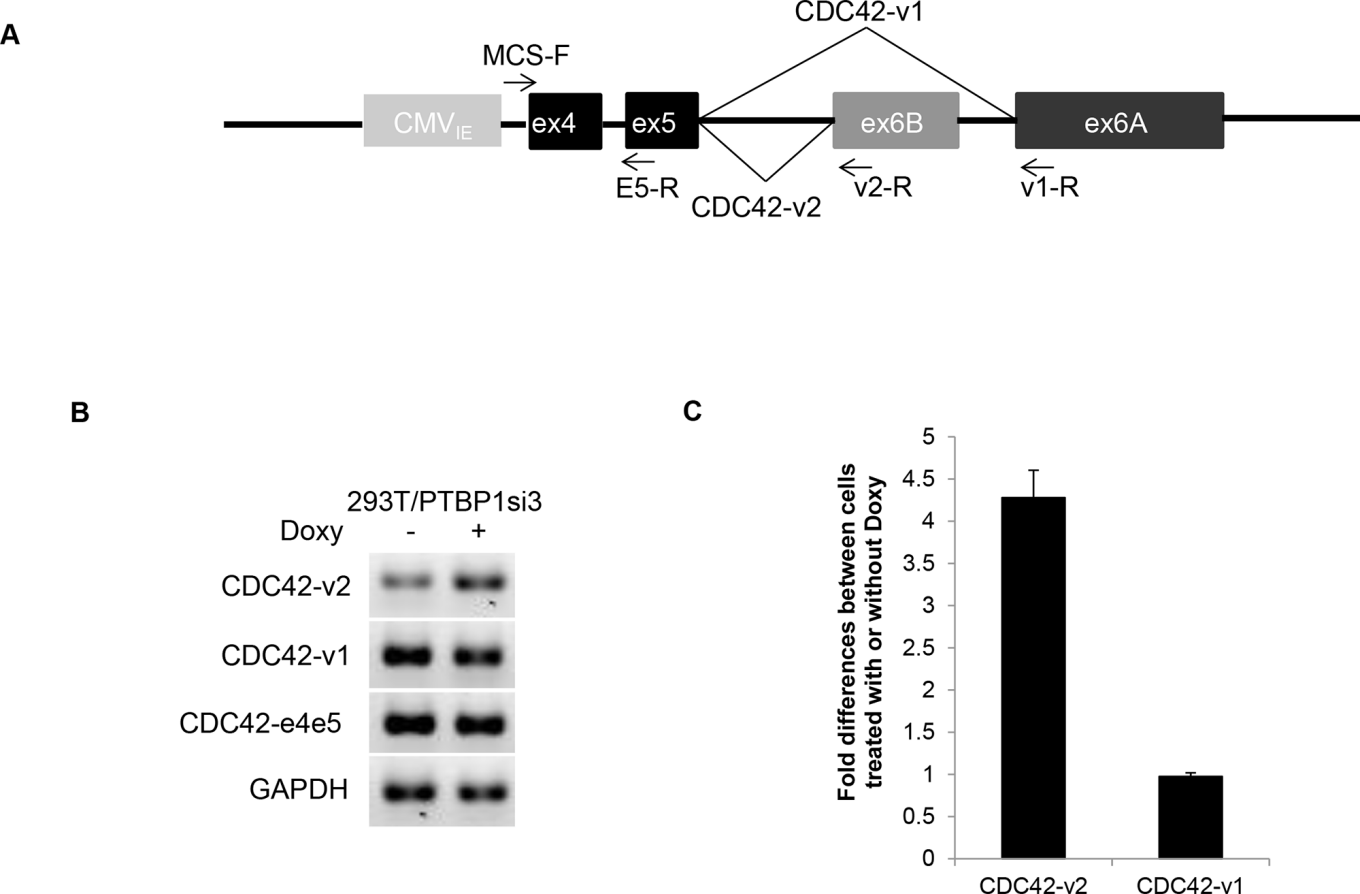
Regulation of CDC42 alternative splicing in minigene by PTBP1 **A.** A diagram of the CDC42 minigene spanning from exon 4 to exon 6A. **B.** Regular RT-PCR showing the expression of CDC42 variants derived from the minigene in 293T/PTBP1si3 cells treated with or without Doxy. **C.** qRT-PCR-determined fold differences of minigene-derived CDC42 variants between 293T/PTBP1si3 cells treated with and without Doxy. Shown are the averages of three independent experiments. Error bar: standard error.

**Figure 4:**
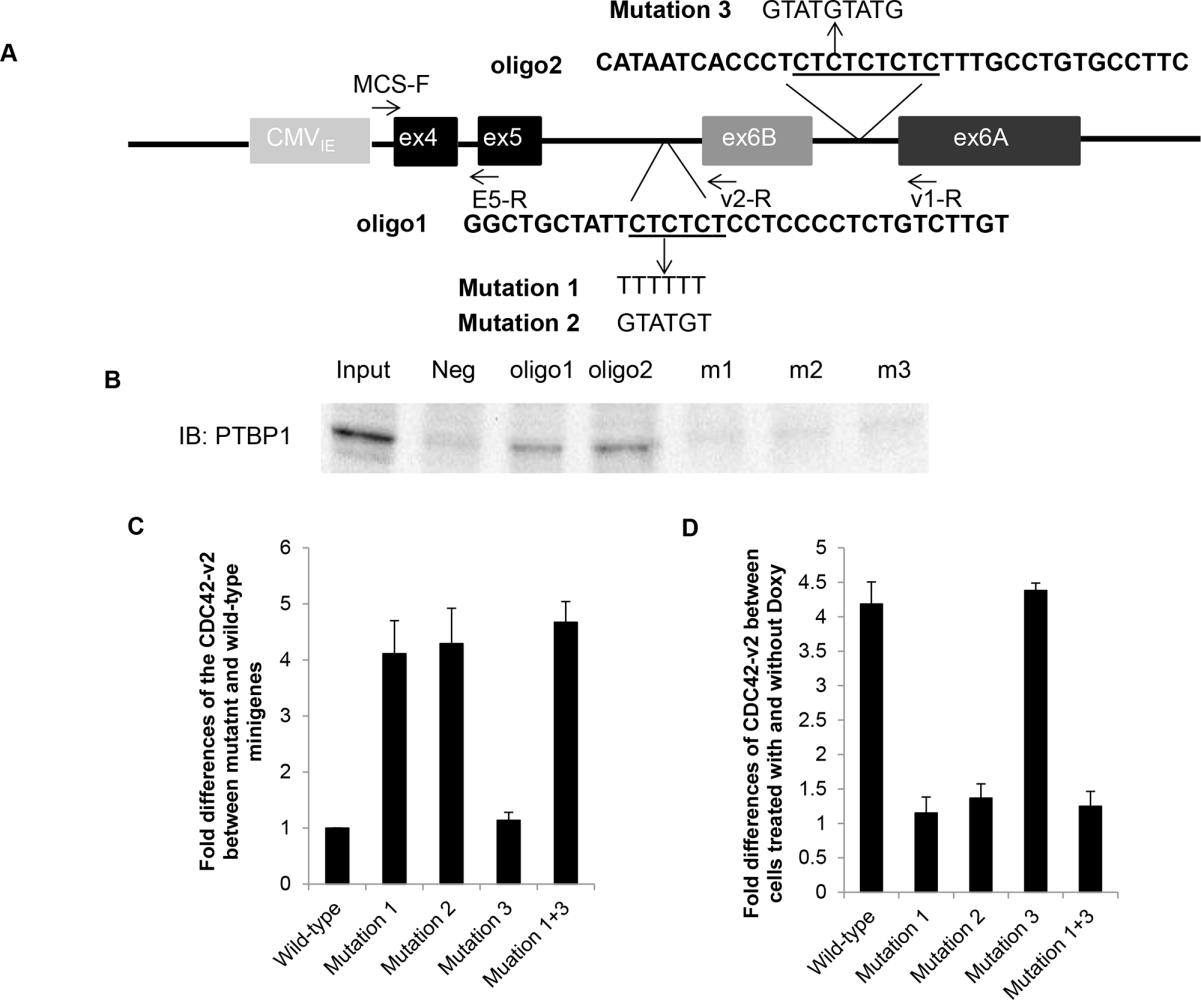
Mutations in PTBP1 binding site on the upstream intron of CDC42 exon 6B relieves repression of exon 6B inclusion by PTBP1 **A.** A diagram showing the mutations introduced into the minigene construct. The underlined sequences are consensus PTBP1 binding sites. **B.** RNA pulldown assay showing the interactions between PTBP1 and RNA oligomers containing consensus PTBP1 binding site (oligo1 and oligo2) or mutated binding site (m1, m2 and m3). **C.** Fold differences of minigene-derived CDC42-v2 expression between 293T/PTBP1si3 cells carrying wild-type minigene and 293T/PTBP1si3 cells carrying mutant minigene grown in the absence of Doxy. **D.** Fold differences of minigene-derived CDC42-v2 expression between 293T/PTBP1si3 cells treated with Doxy and 293T/PTBP1si3 cells treated without Doxy. All fold differences were determined by qRT-PCR. Shown in C and D are the average of three independent experiments. Error bar: standard error.

In order to determine whether PTBP1 is directly involved in the regulation of exon 6B splicing, we conducted minigene analysis. The minigene construct contains the human genomic sequence of CDC42 spanning from exon 4 to exon 6A whose expression is driven by CMV immediate early promoter, as shown in Figure 3A. We first examined whether the splicing of exon 6B in the minigene was regulated by PTBP1. 293T cells engineered to express Doxy-induced PTBP1 siRNA3 (293T/PTBP1si3, Supplementary Figure S1A) were transfected with the minigene construct and then treated with or without Doxy for three days before total RNAs were extracted. We used 293T cells for this experiment because this cell line could be transfected at high efficiency by calcium phosphate precipitation method [22]. The engineered 293T cells exhibited the similar changes in filopodia formation after PTBP1 knockdown, as shown in Supplementary Figure S1B. RT-PCR and qPCR were performed to determine the expression of splice variants derived from the minigene. CDC42-v1 was amplified with primer pair MCS-F and v1-R while CDC42-v2 was amplified with primer pair MCS-F and v2-R. The common primer MCS-F is located in the multiple cloning site of the vector. As shown in Figure 3B and 3C, the splicing of exon 6B in minigene indeed increased about four folds after PTBP1 knockdown while the splicing of exon 6A was slightly reduced. The splicing between exon 4 and exon 5 (CDC42-e4e5) was basically unchanged. Next, we examined the effect of mutations of the PTBP1 consensus binding site CUCUCU [23] in the flanking introns of exon 6B on the splicing of this exon. The CTCTCT in the upstream intron in the minigene (nucleotides -25 to -20 upstream of 3′ splice site) was mutated to TTTTTT (mutation 1) or GTATGT (mutation 2) and the CTCTCTCTC in the downstream intron (nucleotides 163 to 171 downstream of polyadenylation site) was mutated to GTATGTATG (mutation 3) (Figure 4A). We performed RNA pulldown assay to determine the interactions between PTBP1 and RNA oligomers derived from the intronic regions with or without mutations in the consensus PTBP1 binding site. As shown in Figure 4B, PTBP1 was indeed pulled down by oligomers containing consensus PTBP1 binding site but not by the mutant oligomers, indicating that the mutations were able to abolish the interactions between PTBP1 and its target RNAs. After transfection of 293T/PTBP1si3 cells with mutant minigene constructs as well as the wild-type minigene construct, the cell cultures were split and treated with or without Doxy for three days before the expression of splice variants derived from the minigenes was analyzed by qPCR. We first compared the expression of minigene-derived CDC42-v2 among cell cultures receiving no Doxy treatment. As shown in Figure 4C, mutation 1, mutation 2 or mutation combination of 1 and 3 (mutation 1+3) increased the expression of CDC42-v2 four to five folds compared to wild-type minigene, while mutation 3 had little effect on the expression of CDC42-v2 compared to wild-type minigene. The expression of minigene-derived CDC42-v1 was not significantly affected by any of these mutations or the mutation combination (data not shown). Comparison of minigene-derived CDC42-v2 between cells treated with and without Doxy is shown in Figure 4D. As can be seen, mutation 1, mutation 2 or mutation 1+3 abolished the upregulation of CDC42-v2 expression induced by PTBP1 knockdown while mutation 3 alone did not change the effect of PTBP1 knockdown on CDC42-v2 expression. Taken together, above results indicate that regulation of exon 6B inclusion by PTBP1 is mediated by PTBP1 binding site on the upstream intron of exon 6B but not by the binding site on the downstream intron.

### Ectopic expression of CDC42-v2 suppresses filopodia formation

**Figure 5:**
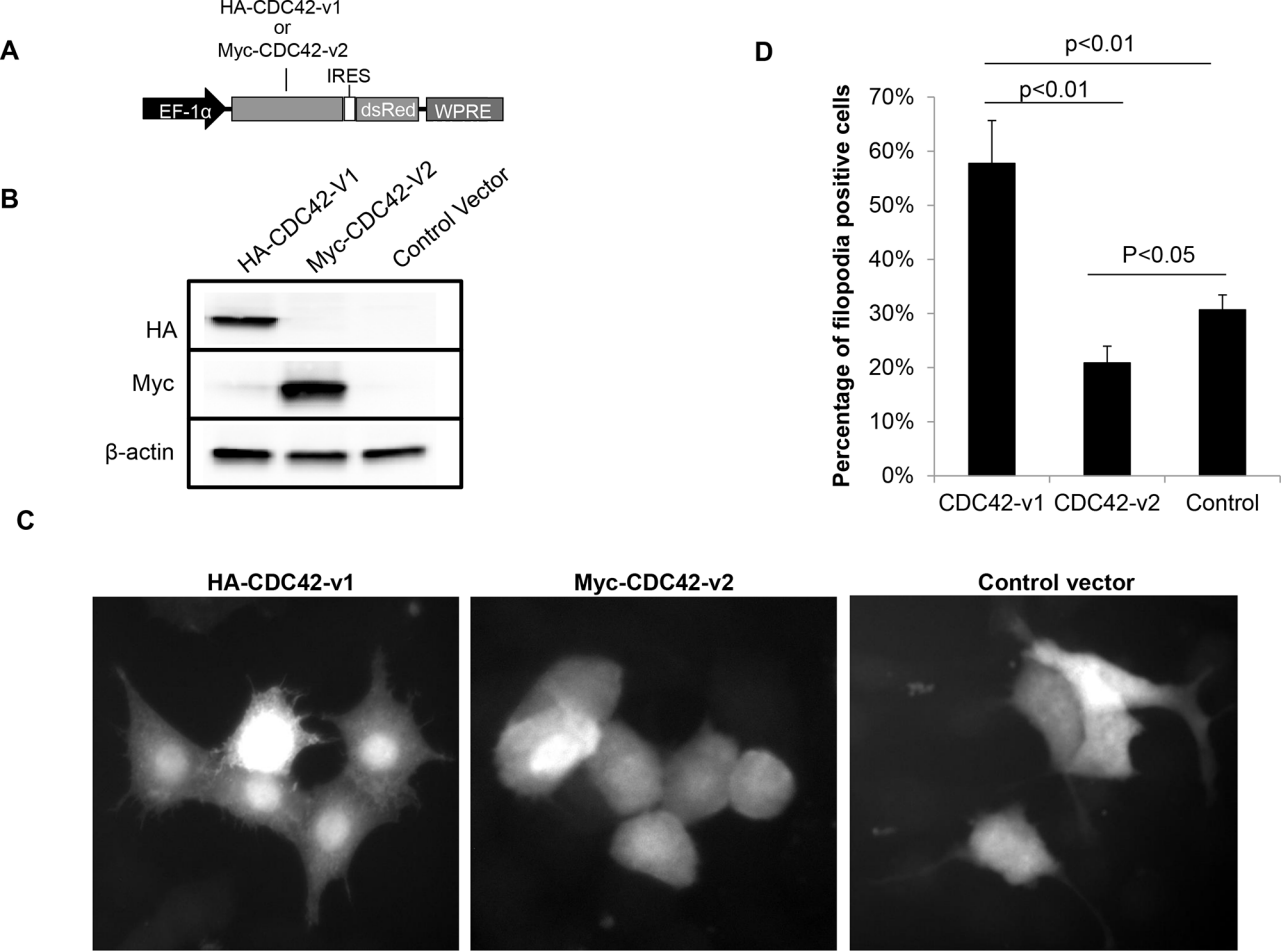
Ectopically expressed CDC42-v2 suppresses filopodia formation **A.** A diagram of the expression cassettes of CDC42 variants in the lentiviral vector. EF-1α: Human elongation factor-1 alpha promoter; IRES: Internal ribosome entry site; WPRE: Woodchuck Hepatitis Virus Posttranscriptional Regulatory Element. **B.** Western blots showing ectopic expression of CDC42 variants. **C.** Micrographs showing the microspikes (filopodia) on the surfaces of NIH3T3 cells expressing CDC42 variants or control vector. **D.** Quantitation of filopodia-positive cells in NIH3T3 cells expressing CDC42 variants or control vector (*n* = 3, error bar: standard error).

To determine whether inhibition of filopodia formation observed in PTBP1 knockdown cells (Figure 1) is mediated by increased expression of CDC42-v2, we examined the effects of ectopically expressed CDC42 splice variants on the formation of filopodia in NIH3T3 cells. The reason for use of NIH3T3 cells instead of A2780 cells was because NIH3T3 cells spread well in the culture and thus allow counting filopodia-positive cells possible, whereas A2780 cells tend to aggregate in the culture and thus make accurate evaluation of filopodia-positive cells difficult. NIH3T3 cells have been widely used for study of filopodia [24]. We infected NIH3T3 cells with lentiviruses carrying HA-CDC42-v1, Myc-CDC42-v2 (Figure 5A) or control vector for two days before evaluating filopodia formation. The ectopic expression of tagged CDC42 variants was confirmed by western blotting, as shown in Figure 5B. The infected cells in the cultures were indicated by red fluorescence of *Discosoma* sp. red fluorescent protein (dsRed). As can be seen in Figure 5C and 5D, cells expressing Myc-CDC42-v2 have fewer spikes than cells infected with control viruses, indicating an inhibitory activity of CDC42-v2 on filopodia formation. In contrast, cells expressing HA-CDC42-v1 have more spikes on the surface than other cells, consistent with previous observation that this CDC42 variant is a positive regulator of filopodia formation [20, 21].

### CDC42-v2 is downregulated in human ovarian cancer cell lines and human ovarian tumors

**Figure 6:**
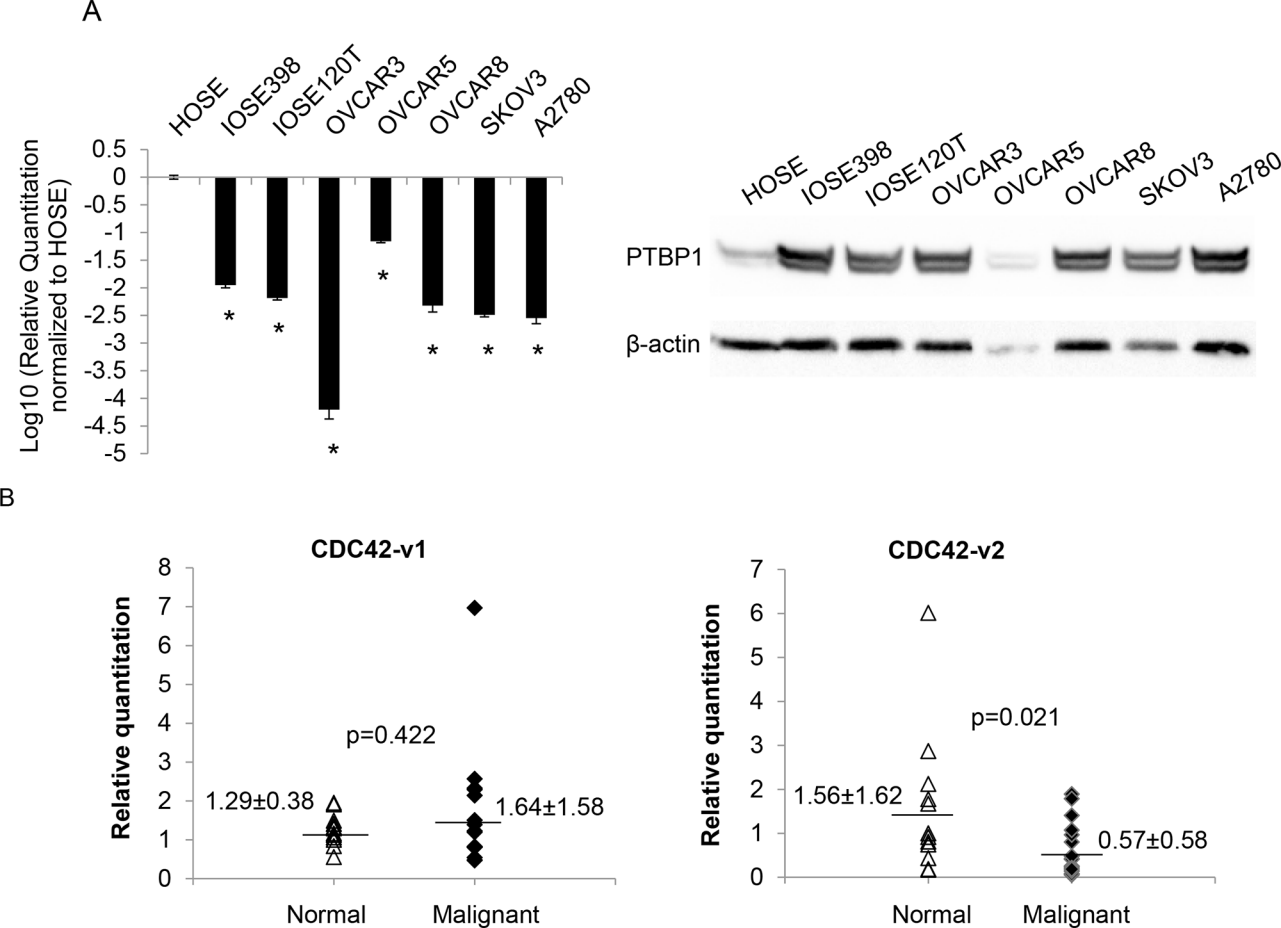
CDC42-v2 is downregulated in ovarian cancer cell lines and ovarian tumor tissues **A.** Left side: Log10-transformed ratios of CDC42-v2 expression in cultured cell lines with HOSE cells as the reference. Shown are the averages of three independent experiments. Error bar: Standard error. *p < 0.01 when compared to HOSE. Right side: PTBP1 expression in these cells. **B.** Expression of CDC42 variants in human ovarian tumor and normal tissues determined by qPCR.

Our previous study showed that PTBP1 was overexpressed in human ovarian tumors and a panel of ovarian cancer cell lines [8]. The observation that CDC42-v2 was upregulated by PTBP1 knockdown, as shown in Figure 2, suggested that this CDC42 variant might be downregulated in ovarian cancer cells. Therefore, we examined its expression by qPCR in two immortalized ovarian surface epithelial cells (IOSE398 and IOSE120T) as well as a panel of ovarian cancer cell lines. Compared to human normal ovarian surface epithelial cells (HOSE), CDC42-v2 was indeed downregulated in these immortalized cells and ovarian cancer cell lines (Figure 6A, left side) but the expression of CDC42-v1 was not significantly different (data not shown). Western blotting confirmed the overexpression of PTBP1 in these cells, as shown on the right side of Figure 6A. We also measured the expression of CDC42 variants by qPCR in 18 normal ovarian tissues and 29 malignant ovarian tumor tissues. As shown in Figure 6B, the expression of CDC42-v2 was lower in the malignant tissues than in the normal tissues, while the differences in the abundance of CDC42-v1between normal and tumor tissues were not statistically significant.

### Effects of ectopic expression of CDC42 splice variants on tumor cell behaviors

**Figure 7:**
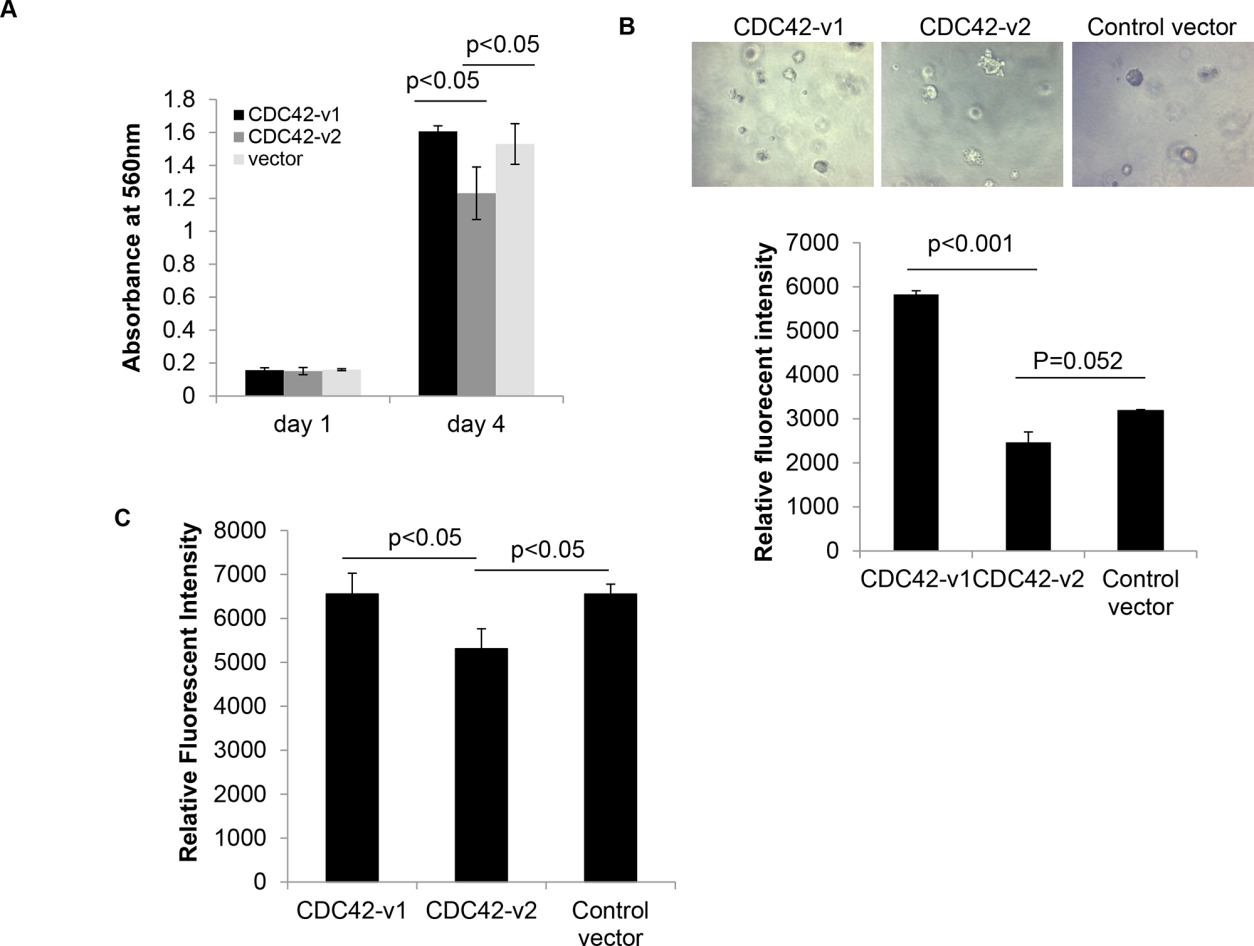
Ectopically expressed CDC42-v2 impairs transformation properties of ovarian cancer cells **A.** MTT assay of tumor cell growth (*n* = 3, error bar: standard error). **B.** Colony formation assay in soft agar. Top panel: Micrographs of colonies formed in soft agar; Bottom panel: Quantitation of colony formation expressed as Relative Fluorescent Intensity (*n* = 3, error bar: standard error). **C.** Quantitation of cell invasiveness expressed as Relative Fluorescent Intensity (*n* = 3, error bar: standard error).

Given the upregulation of CDC42-v2 in PTBP1-knockdown tumor cells, which were showed in our previous study to have inhibited cell growth and impaired transformation properties [8], and decreased expression of this variant in a panel of ovarian cancer cell lines and ovarian tumor tissues (Figures 6A and 6B), we wondered whether CDC42-v2 had any antitumor activity and could mediate the antitumor effects of PTBP1 knockdown on tumor cells. To answer these questions, we first examined whether ectopic expression of CDC42-v2 affected ovarian tumor cell behaviors. As shown in Figure 7A, A2780 cells expressing Myc-CDC42-v2 grew slower compared to cells expressing HA-CDC42-v1 or cells carrying the control vector while there was not a statistically significant difference between the latter two cell cultures. Similarly, we also observed inhibited invasive activity in A2780 cells expressing Myc-CDC42-v2 compared to other two cell cultures (Figure 7C). In regard to colony formation in soft agar, although Myc-CDC42-v2 reduced colony formation of A2780 cells compared to the control vector, the difference between two was not statistically significant (Figure 7B). In contrast, CDC42-v1 enhanced A2780 cells’ capability to form colonies in soft agar compared to the control vector (Figure 7B). Taken together, these results indicate that two CDC42 variants have different effects on tumor cell behavior. Similar results were obtained with another ovarian cancer cell line, SKOV3, in these assays (Supplementary Figure S2), indicating they are not cell line-specific.

To determine whether upregulated CDC42-v2 mediated the antitumor effects of PTBP1 knockdown, we tried to suppress its expression using siRNAs targeting the unique sequence of this variant. Unfortunately, none of the tested siRNAs could effectively suppress the expression of CDC42-v2 at mRNA level, as determined by qPCR. To definitively address this issue, we will need to knock out CDC42-v2-specific exon, exon 6B, using gene targeting technology such as CRISPR/Cas9 system and then examine the effects on tumor cell behaviors. We are currently conducting these experiments and will report the results in the future.

## DISCUSSION

PTBP1 is an RNA binding protein involved in multiple steps of post-transcriptional regulation of gene expression and best known for its function in alternative splicing [25]. Through its regulated genes, PTBP1 has been implicated in the control of several cellular activities such as cell differentiation and cell metabolism [26–28]. In the present study, we observed for the first time that PTBP1 was also important for the formation of filopodia (see Figure 1), suggesting a new role for this splicing factor. Filopodia are the long, slender cellular protrusions formed of tightly bundled actin filaments [29]. They are involved in several important cellular processes, such as cell migration [30] and transport of signaling proteins [31], and hence are essential for normal development, wound healing and signaling transduction. Moreover, there is accumulating evidence indicating that filopodia play a part in tumor development and progression. For example, Fascin, the major actin bundling protein found in the filopodia [32], was upregulated during colorectal carcinogenesis and exclusively localized at the invasive front of tumor tissues [33]. Increased expression of Fascin was correlated with poor prognosis of several carcinomas including ovarian cancer [34–37]. Recently, Shibue and colleagues demonstrated that the formation of filopodium-like protrusions was a necessary step for the development of macroscopic metastasis in the lung by breast cancer cells [38]. Combining with these studies, our observation that filopodia formation is inhibited by PTBP1 knockdown suggests that PTBP1's role in tumorigenesis, the finding of our previous work [8], is probably partly mediated by filopodia.

CDC42 is an important positive regulator of filopodia formation, which induces filopodia by interacting with IRSp53, a membrane deforming protein, to switch it from an inhibitor to an activator of actin assembly [20, 21]. CDC42 has two isoforms. In this report, we show that the generation of CDC42 isoforms is regulated by PTBP1, which represses the inclusion of the alternative terminal exon 6B. We also show that the PTBP1 binding site in the upstream flanking intron is critical to mediate this repression whereas the binding site in the downstream intron is not. This observation is consistent with previous finding that PTBP1 acts as a splicing repressor when binding to upstream intron or within the cassette exon to be skipped [39].

Previously, it was reported that PTBP1 could regulate the inclusion of alternative terminal exon at the level of polyadenylation, as in the case of CT/CGRP gene [40, 41]. Although we cannot exclude this mechanism in the regulation of CDC42 exon 6B inclusion, we do not think it plays an important role. As shown in the Supplementary Figure S3, exons 6A and 6B carry similar polyadenylation signals, including AUUAAA hexamer, which is the more frequent variant of the canonical AAUAAA hexamer [42], and the U/GU-rich downstream sequence element (DSE). This similarity suggests that both terminal exons would have similar efficiency for polyadenylation and PTBP1 could not differentially regulate their polyadenylation by competing with the 3′ end processing factors [43]. Therefore, if PTBP1 regulated the inclusion of exon 6B at the level of polyadenylation, this regulation would be very likely mediated by its interaction with the *cis*-element further downstream of DSE of exon 6B. However, as discussed above, disruption of this interaction by mutation 3 had little effect on the inclusion of exon 6B, arguing against an active involvement of polyadenylation in the regulation of exon 6B inclusion.

The functional differences of two CDC42 isoforms are largely unknown. In this report, we found that CDC42-v2 had differing activities than CDC42-v1 in regulating filopodia formation, which was suppressed by ectopically expressed CDC42-v2 but promoted by ectopically expressed CDC42-v1 (Figure 5). These results suggest that PTBP1 knockdown-induced inhibition of filopodia formation may be partly mediated by the upregulation of CDC42-v2. It is possible that there are other factors contributing to this inhibitory effect. One example is PTBP2, a paralog of PTBP1, which Cheung et al. reported to play an inhibitory role in filopodia formation [44]. Previous studies showed that the expression of PTBP2 was upregulated when PTBP1 was downregulated [45, 46]. In our study, we observed a substantial increase of PTBP2 expression in the PTBP1-knockdown cells, as shown in the Supplementary Figure S4. Therefore, the inhibition of filopodia formation induced by PTBP1 knockdown may also be partly mediated by increased PTBP2.

It is worth noting that our observation is contrary to a previous report [47], which showed that CDC42-v2 was able to stimulate filopodia formation just like CDC42-v1. The discrepancy between the two studies is probably due to the use of different cultured cells and different expression constructs. In our study, we used Myc-tagged wild-type CDC42-v2 while the other group used GFP-tagged constitutively active mutant CDC42-v2. It was noted before that activated mutant Ras might have significantly different subcellular distribution than endogenous counterparts and thus might not induce the same phenotypes as the wild-type isoforms do [48]. Given the high similarity between CDC42 proteins and Ras proteins in their molecular functions and regulation, it is likely that constitutively active CDC42 mutants might also function differently than their wild-type counterparts.

Another functional difference we observed between two CDC42 isoforms is that CDC42-v2 had certain antitumor activities which were not found with CDC42-v1. As mentioned above, previous studies revealed a complex role for CDC42 in tumorigenesis: On one hand, CDC42 was implicated in almost every step of tumorigenic process, promoting neoplastic transformation, tumor invasion and metastasis [13]; on the other hand, knockout of CDC42 resulted in tumor formation or partial transformation [16, 17], suggesting that CDC42 is a tumor suppressor. It has remained unclear what mechanism contributes to this paradoxical phenomenon. Our observation reported here suggests a hypothesis that the tumor promoting activities of CDC42 come from CDC42-v1 while the tumor suppressor activities come from CDC42-v2. This is because all studies showing CDC42 as an oncoprotein have been conducted with CDC42-v1 or its mutants and thus CDC42's tumor promoting function can be attributed to this variant. In gene knockout studies, however, both CDC42 variants were depleted and the elimination of CDC42-v2 removed a suppression of tumorigenesis and thus led to transformation. To definitively determine the roles of each CDC42 variant in normal physiology and pathological processes, it would be necessary to specifically knock out each variant in cultured cells and/or in mice and then examine the resulting phenotypes.

## MATERIALS AND METHODS

### Cell lines and human tissue specimens

Sublines of A2780 ovarian cancer cell line, A2780/PTBP1si1, A2780/PTBP1si3 and A2780/LUCsi, were established in our previous study [8]. These subline cells express doxycycline (Doxy)-induced PTBP1 siRNA1, siRNA3 and luciferase siRNA, respectively. 293T/PTBP1si3 cells were established as described in [8], which express Doxy-induced PTBP1 siRNA3. All the cell cultures used in this study were grown in Dulbecco's Modified Eagle Medium (DMEM) supplemented with 10% fetal bovine serum at 37°C, 5% CO_2_. Human normal ovarian surface epithelial cells (HOSE) and immortalized HOSE cells (IOSE398 and IOSE120T) were a gift from Dr. Nelly Auersperg [49]. Snap-frozen human ovarian tumor and normal tissue blocks were obtained from The Cooperative Human Tissue Network, Midwestern Division (Columbus, OH). Institutional Review Board of the University of Illinois at Chicago approved use of these human specimens in this study (protocol #: 20100036).

### Filopodia detection

Cells grown on glass coverslips were fixed with 4% paraformaldehyde, pH 7.0 for 10 minutes, followed by permeabilization with 0.5% Triton X-100 for 5 minutes before they were stained with 200 ul of 100 nM rhodamine phalloidin (Cytoskeleton, Inc., Denver, CO) at room temperature in the dark for 30 minutes. The cells were washed briefly in 1xPBS between incubations. The stained coverslips were mounted on the glass slides using VECTASHIELD^®^ Mounting Media with DAPI (Vector Laboratories, Inc, Burlingame, CA). For filopodia detection in NIH3T3 cells, cells were examined under microscope for microspikes on the cell surface without phalloidin staining. A cell was counted as filopodia positive if five or more microspikes were present on its surface [24].

### PCR

Total RNAs were extracted with Trizol reagent (Invitrogen, Carlsbad, CA) from cultured cells or human frozen tissues by following the manufacturer's instruction. cDNA was synthesized from 2 μg of total RNA with High Capacity cDNA Reverse Transcription Kit (Applied Biosystems, Foster City, CA). Regular PCR was carried out with Phire Hot Start II DNA polymerase (Thermo Fisher Scientific, Waltham, MA) and qPCR was set up with Fast SYBR Green Master Mix (Applied Biosystems) and run in StepOne Plus Real-Time PCR System (Applied Biosystems). Comparative C_T_ (ΔΔ C_T_) method was used to determine the relative quantitation of transcript levels [50] with GAPDH as the endogenous control. For comparison of minigene-derived CDC42 variants expression, the expression of CDC42 fragment spanning exons 4 and 5 (CDC42-e4e5) was used as the control to normalize the transfection efficiency. The primer pairs for amplification of endogenous CDC42-v1 and CDC42-v2 were common forward primer E5-F, 5′-AGGCTGTCAAGTATGTGGAG-3′, coupled with CDC42-v1 specific reverse primer v1-R, 5′-ACAGAGGTTGCTCTAAGGTG-3′, or CDC42-v2 specific reverse primer v2-R, 5′-TCATAGCA GCACACACCTGC-3′ (Figure 2A). The primer pairs for amplification of CDC42-v1 and CDC42-v2 derived from minigenes were common forward primer MCS-F, 5′-GCTAGCGCTACCGGACTCAGAT-3′, which is located in the multiple cloning site of minigene plasmids (Figure 3A), coupled with v1-R or v2-R. The primer pair for amplification of CDC42-e4e5 was MCS-F and E5-R, 5′-GGTGAG TTATCTCAGGCACCC-3′ (Figure 3A).

### Preparation of CDC42 minigene constructs

The genomic sequence of human CDC42 spanning exon 4 to the last exon (exon 6A) was PCR-amplified using the genomic DNA isolated from A2780 cells as the template. The PCR product was then cloned into the pEGFP-N1 vector (Promega, Madison, WI) between Age I and Not I sites to replace the coding sequence of EGFP. To mutate the PTBP1 binding sites on the minigene, we used overlap extension PCR method as described in [51]. Briefly, to introduce Mutation 1 (Figure 4A) into the minigene, we first generated two overlapping PCR products using primer pair Bpu10i-F and m1-R and primer pair m1-F and AclI-R. Bpu10i-F, 5′-AGTTTCTGGCTGAGGTGTAAG-3′, is located upstream of the mutation and carries Bpu10i site (underlined sequence); m1-R, 5′-**AAAAAAAATAG CAGCCAGGTTAGAGG A**-3′, and m1-F, **5′**-**TCCTCTA ACC TGGCTGCTATTTTTTTT**CCTCCCCTCTGTCT TGTAG A-3′, carry the Mutation 1 (underlined sequences) and overlap with each other (sequences in bold); AclI-R, 5′-AACTCAAGCAGCAGAACGTTA-3′, is located downstream of the mutation and carries AclI site. Both Bpu10i and AclI are single cutters of the minigene. The generated PCR products were gel-purified and then used for overlap extension PCR with primer pair Bpu10i-F and AclI-R. The resulting PCR product was subsequently cloned into the minigene plasmid between Bpu10i and AclI sites. The Mutation 2 and Mutation3 were introduced into the minigene in the similar way. These mutations were confirmed by DNA sequencing as shown in Supplementary Figure S5.

### Minigene assay

The assay was carried out with the 293T subline cells 293T/PTBP1si3, which was established to express Doxy-induced PTBP1 siRNA3 by the procedure as described in our previous study [8]. The induction of PTBP1 knockdown was confirmed by western blotting, as shown in Supplementary Figure S1A. The minigene constructs were introduced into 293T/PTBP1si3 cells by calcium phosphate precipitation transfection [22]. Twenty-four hours after transfection, the cells were split and treated with or without Doxy at 0.1 μg/μl for three days before total RNAs were extracted. The expression of CDC42 variants was analyzed by RT-PCR and qPCR, as described above.

### RNA pulldown assay

Five biotin-labeled RNA oligonucleotides were synthesized by Integrated DNA Technologies (Skokie, IL). These RNA oligomers are: oligo1, 5′-biotin-GGCUGCUAUUCUCUCUCCUCCCCUCUGUCUU GU-3′, which is derived from the upstream intronic sequence of exon 6B; oligo2, 5′-biotin-CAUAAUCACCCUCUCUCUCUCUUUGCCUGUGCCUUC-3′, which is derived from the downstream intronic sequence of exon 6B; m1 and m2 are the mutants of the oligo1 with the underlined sequence mutated to UUUUUU and GUAUGU, respectively; m3 is the mutant of oligo2 with the underlined sequence mutated to GUAUGUAUG. The pulldown assay was carried out using Pierce™ Magnetic RNA-Protein Pull-Down Kit (Life Technology, Grand Island, NY). The PTBP1 was pulled down from the nuclear extracts of 293T cells, which were prepared as previously described [52].

### Ectopic expression of CDC42 splice variants

The coding sequences for CDC42-v1 and CDC42-v2 were amplified from cDNAs of A2780 cells and cloned into pCMV-HA and pCMV-Myc vectors (Clontech Laboratories, Inc., Mountain View, CA), respectively, between EcoR I and Kpn I sites. The resulting plasmids were then used as the templates to amplify HA-CDC42-v1 and Myc-CDC42-v2, respectively, which were subsequently cloned into the lentiviral vector pLV-tTRKRAB-red [53] to replace the coding sequence of tTRKRAB. The resulting lentiviral plasmids carry an expression cassette of HA-CDC42-v1 or Myc-CDC42-v2 coding sequence coupled with dsRed coding sequence by internal ribosomal entry site (IRES) (Figure 5A). The lentiviruses were prepared as described previously [8].

### Western blotting

Cells were washed once with 1xPBS before lysed with 1x Laemmli sample buffer (60 mM Tris pH 6.8, 2%SDS, 10% glycerol, 5% β-mecaptoethanol and 0.002% bromphenol blue). The cell lysates were then sonicated and boiled for 5 minutes before used for western blotting, which was performed as described previously [8]. The antibodies recognizing HA tag (Cat. #: sc-7392) and Myc tag (Cat. #: sc-40) were purchased from Santa Cruz Biotechnology (Dallas, TX).

### Cell growth curve

A2780 cells infected with CDC42 variant-expressing lentiviruses or control lentiviruses were seeded at a density of 1000 cells per well in quadruplicate. On day 1 and day 4 after seeding, viable cells were evaluated by MTT assay as described previously [8].

### Colony formation assay

This assay was performed in 96-well plates using CytoSelect™ 96-Well Cell Transformation Assay kit (Cell Biolabs, Inc., San Diego, CA), according to the manufacturer's instructions. For each cell line, 7 × 10^3^ cells mixed in the 0.4% agar solution were seeded per well in triplicate on the top of solidified 0.6% base agar. After incubation at 37°C and 5% CO2 for eight days, cell colonies was examined under a microscope and quantitated by the provided CyQuant GR dye following the kit's manual.

### *In vitro* invasion assay

The invasiveness of tumor cells was examined using CytoSelect™ 96-Well Cell Invasion Assay kit (Cell Biolabs, Inc.) according to the manufacturer's instructions. Briefly, 5 × 10^4^ cells in 0.1 ml of serum-free DMEM were seeded in triplicate into the rehydrated basement membrane inserts, which were placed in the 96-well plate containing 0.15 ml per well of DMEM supplemented with 10% FBS. After incubation at 37°C and 5% CO2 for 24 hours, the invaded cells on the bottom of the membrane were detached and quantitated by CyQuant GR dye as described in the kit's manual.

### Statistical analysis

Student's *t*-test was used to determine the statistical significances of comparisons. All tests were two-sided and *p*-values ≤ 0.05 was considered significant.

## SUPPLEMENTARY FIGURES



## References

[1] Kalsotra A, Cooper T.A (2011). Functional consequences of developmentally regulated alternative splicing. Nat Rev Genet.

[2] Wang G.S, Cooper T.A (2007). Splicing in disease: disruption of the splicing code and the decoding machinery. Nat Rev Genet.

[3] Yoshida K, Sanada M, Shiraishi Y, Nowak D, Nagata Y, Yamamoto R, Sato Y, Sato-Otsubo A, Kon A, Nagasaki M, Chalkidis G, Suzuki Y, Shiosaka M (2011). Frequent pathway mutations of splicing machinery in myelodysplasia. Nature.

[4] Venables J.P, Klinck R, Koh C, Gervais-Bird J, Bramard A, Inkel L, Durand M, Couture S, Froehlich U, Lapointe E, Lucier J.F, Thibault P, Rancourt C (2009). Cancer-associated regulation of alternative splicing. Nat Struct Mol Biol.

[5] Karni R, de Stanchina E, Lowe S.W, Sinha R, Mu D, Krainer A.R (2007). The gene encoding the splicing factor SF2/ASF is a proto-oncogene. Nat Struct Mol Biol.

[6] Sjoblom T, Jones S, Wood L.D, Parsons D.W, Lin J, Barber T.D, Mandelker D, Leary R.J, Ptak J, Silliman N, Szabo S, Buckhaults P, Farrell C (2006). The consensus coding sequences of human breast and colorectal cancers. Science.

[7] Golan-Gerstl R, Cohen M, Shilo A, Suh S.S, Bakacs A, Coppola L, Karni R (2011). Splicing factor hnRNP A2/B1 regulates tumor suppressor gene splicing and is an oncogenic driver in glioblastoma. Cancer Res.

[8] He X, Pool M, Darcy K.M, Lim S.B, Auersperg N, Coon J.S, Beck W.T (2007). Knockdown of polypyrimidine tract-binding protein suppresses ovarian tumor cell growth and invasiveness *in vitro*. Oncogene.

[9] He X, Arslan A.D, Ho T.T, Yuan C, Stampfer M.R, Beck W.T (2014). Involvement of polypyrimidine tract-binding protein (PTBP1) in maintaining breast cancer cell growth and malignant properties. Oncogenesis.

[10] He X, Arslan A.D, Pool M.D, Ho T.T, Darcy K.M, Coon J.S, Beck W.T (2011). Knockdown of splicing factor SRp20 causes apoptosis in ovarian cancer cells and its expression is associated with malignancy of epithelial ovarian cancer. Oncogene.

[11] Cerione R.A (2004). Cdc42: new roads to travel. Trends Cell Biol.

[12] Sinha S, Yang W (2008). Cellular signaling for activation of Rho GTPase Cdc42. Cell Signal.

[13] Stengel K, Zheng Y (2011). Cdc42 in oncogenic transformation, invasion, and tumorigenesis. Cell Signal.

[14] Fidyk N, Wang J.B, Cerione R.A (2006). Influencing cellular transformation by modulating the rates of GTP hydrolysis by Cdc42. Biochemistry.

[15] Johnson E, Seachrist D.D, DeLeon-Rodriguez C.M, Lozada K.L, Miedler J, Abdul-Karim F.W, Keri R.A (2010). HER2/ErbB2-induced breast cancer cell migration and invasion require p120 catenin activation of Rac1 and Cdc42. J Biol Chem.

[16] van Hengel J, D'Hooge P, Hooghe B, Wu X, Libbrecht L, De Vos R, Quondamatteo F, Klempt M, Brakebusch C, van Roy F (2008). Continuous cell injury promotes hepatic tumorigenesis in cdc42-deficient mouse liver. Gastroenterology.

[17] Yang L, Wang L, Kalfa T.A, Cancelas J.A, Shang X, Pushkaran S, Mo J, Williams D.A, Zheng Y (2007). Cdc42 critically regulates the balance between myelopoiesis and erythropoiesis. Blood.

[18] Nishimura A, Linder M.E (2013). Identification of a novel prenyl and palmitoyl modification at the CaaX motif of Cdc42 that regulates RhoGDI binding. Mol Cell Biol.

[19] Wirth A, Chen-Wacker C, Wu Y.W, Gorinski N, Filippov M.A, Pandey G, Ponimaskin E (2013). Dual lipidation of the brain-specific Cdc42 isoform regulates its functional properties. Biochem J.

[20] Disanza A, Bisi S, Winterhoff M, Milanesi F, Ushakov D.S, Kast D, Marighetti P, Romet-Lemonne G, Muller H.M, Nickel W, Linkner J, Waterschoot D, Ampe C (2013). CDC42 switches IRSp53 from inhibition of actin growth to elongation by clustering of VASP. EMBO J.

[21] Krugmann S, Jordens I, Gevaert K, Driessens M, Vandekerckhove J, Hall A (2001). Cdc42 induces filopodia by promoting the formation of an IRSp53:Mena complex. Curr Biol.

[22] Kingston R.E, Chen C.A, Rose J.K Calcium phosphate transfection. Curr Protoc Mol Biol.

[23] Oberstrass F.C, Auweter S.D, Erat M, Hargous Y, Henning A, Wenter P, Reymond L, Amir-Ahmady B, Pitsch S, Black D.L, Allain F.H (2005). Structure of PTB bound to RNA: specific binding and implications for splicing regulation. Science.

[24] Gadea G, Lapasset L, Gauthier-Rouviere C, Roux P (2002). Regulation of Cdc42-mediated morphological effects: a novel function for p53. EMBO J.

[25] Kafasla P, Mickleburgh I, Llorian M, Coelho M, Gooding C, Cherny D, Joshi A, Kotik-Kogan O, Curry S, Eperon I.C, Jackson R.J, Smith C.W (2012). Defining the roles and interactions of PTB. Biochem Soc Trans.

[26] Zheng S, Gray E.E, Chawla G, Porse B.T, O'Dell T.J, Black D.L (2012). PSD-95 is post-transcriptionally repressed during early neural development by PTBP1 and PTBP2. Nat Neurosci.

[27] David C.J, Chen M, Assanah M, Canoll P, Manley J.L (2010). HnRNP proteins controlled by c-Myc deregulate pyruvate kinase mRNA splicing in cancer. Nature.

[28] Clower C.V, Chatterjee D, Wang Z, Cantley L.C, Vander Heiden M.G, Krainer A.R (2010). The alternative splicing repressors hnRNP A1/A2 and PTB influence pyruvate kinase isoform expression and cell metabolism. Proc Natl Acad Sci U S A.

[29] Mattila P.K, Lappalainen P (2008). Filopodia: molecular architecture and cellular functions. Nat Rev Mol Cell Biol.

[30] Ridley A.J (2011). Life at the leading edge. Cell.

[31] Sanders T.A, Llagostera E, Barna M (2013). Specialized filopodia direct long-range transport of SHH during vertebrate tissue patterning. Nature.

[32] Vignjevic D, Kojima S, Aratyn Y, Danciu O, Svitkina T, Borisy G.G (2006). Role of fascin in filopodial protrusion. J Cell Biol.

[33] Vignjevic D, Schoumacher M, Gavert N, Janssen K.P, Jih G, Lae M, Louvard D, Ben-Ze'ev A, Robine S (2007). Fascin, a novel target of beta-catenin-TCF signaling, is expressed at the invasive front of human colon cancer. Cancer Res.

[34] Puppa G, Maisonneuve P, Sonzogni A, Masullo M, Chiappa A, Valerio M, Zampino M.G, Franceschetti I, Capelli P, Chilosi M, Menestrina F, Viale G, Pelosi G (2007). Independent prognostic value of fascin immunoreactivity in stage III-IV colonic adenocarcinoma. Br J Cancer.

[35] Pelosi G, Pastorino U, Pasini F, Maissoneuve P, Fraggetta F, Iannucci A, Sonzogni A, De Manzoni G, Terzi A, Durante E, Bresaola E, Pezzella F, Viale G (2003). Independent prognostic value of fascin immunoreactivity in stage I nonsmall cell lung cancer. Br J Cancer.

[36] Yoder B.J, Tso E, Skacel M, Pettay J, Tarr S, Budd T, Tubbs R.R, Adams J.C, Hicks D.G (2005). The expression of fascin, an actin-bundling motility protein, correlates with hormone receptor-negative breast cancer and a more aggressive clinical course. Clin Cancer Res.

[37] Daponte A, Kostopoulou E, Papandreou C.N, Daliani D.D, Minas M, Koukoulis G, Messinis I.E (2008). Prognostic significance of fascin expression in advanced poorly differentiated serous ovarian cancer. Anticancer Res.

[38] Shibue T, Brooks M.W, Inan M.F, Reinhardt F, Weinberg R.A (2012). The outgrowth of micrometastases is enabled by the formation of filopodium-like protrusions. Cancer Discov.

[39] Llorian M, Schwartz S, Clark T.A, Hollander D, Tan L.Y, Spellman R, Gordon A, Schweitzer A.C, de la Grange P, Ast G, Smith C.W (2010). Position-dependent alternative splicing activity revealed by global profiling of alternative splicing events regulated by PTB. Nat Struct Mol Biol.

[40] Lou H, Helfman D.M, Gagel R.F, Berget S.M (1999). Polypyrimidine tract-binding protein positively regulates inclusion of an alternative 3′-terminal exon. Mol Cell Biol.

[41] Lou H, Gagel R.F, Berget S.M (1996). An intron enhancer recognized by splicing factors activates polyadenylation. Genes Dev.

[42] Millevoi S, Vagner S (2010). Molecular mechanisms of eukaryotic pre-mRNA 3′ end processing regulation. Nucleic Acids Res.

[43] Castelo-Branco P, Furger A, Wollerton M, Smith C, Moreira A, Proudfoot N (2004). Polypyrimidine tract binding protein modulates efficiency of polyadenylation. Mol Cell Biol.

[44] Cheung H.C, Hai T, Zhu W, Baggerly K.A, Tsavachidis S, Krahe R, Cote G.J (2009). Splicing factors PTBP1 and PTBP2 promote proliferation and migration of glioma cell lines. Brain.

[45] Boutz P.L, Stoilov P, Li Q, Lin C.H, Chawla G, Ostrow K, Shiue L, Ares M, Black D.L (2007). A post-transcriptional regulatory switch in polypyrimidine tract-binding proteins reprograms alternative splicing in developing neurons. Genes Dev.

[46] Spellman R, Llorian M, Smith C.W (2007). Crossregulation and functional redundancy between the splicing regulator PTB and its paralogs nPTB and ROD1. Mol Cell.

[47] Kang R, Wan J, Arstikaitis P, Takahashi H, Huang K, Bailey A.O, Thompson J.X, Roth A.F, Drisdel R.C, Mastro R, Green W.N, Yates J.R, Davis N.G (2008). Neural palmitoyl-proteomics reveals dynamic synaptic palmitoylation. Nature.

[48] Karnoub A.E, Weinberg R.A (2008). Ras oncogenes: split personalities. Nat Rev Mol Cell Biol.

[49] Maines-Bandiera S.L, Kruk P.A, Auersperg N (1992). Simian virus 40-transformed human ovarian surface epithelial cells escape normal growth controls but retain morphogenetic responses to extracellular matrix. Am J Obstet Gynecol.

[50] Schmittgen T.D, Livak K.J (2008). Analyzing real-time PCR data by the comparative C(T) method. Nat Protoc.

[51] Nelson M.D, Fitch D.H (2011). Overlap extension PCR: an efficient method for transgene construction. Methods Mol Biol.

[52] Nilsen T.W (2013). Preparation of Nuclear Extracts from HeLa cells. Cold Spring Harb Protoc.

[53] Wiznerowicz M, Trono D (2003). Conditional suppression of cellular genes: lentivirus vector-mediated drug-inducible RNA interference. J Virol.

