# Supplementary material for: Regulation and functional significance of CDC42 alternative splicing in ovarian cancer
Source: Oncotarget. 2015 Aug 22;6(30):29651–63. doi: 10.18632/oncotarget.4865 (PMC4745753; doi:10.18632/oncotarget.4865)
Supplement: Supplementary file 1 [file oncotarget-06-29651-s001.pdf]

SUPPLEMENTARY FIGURES

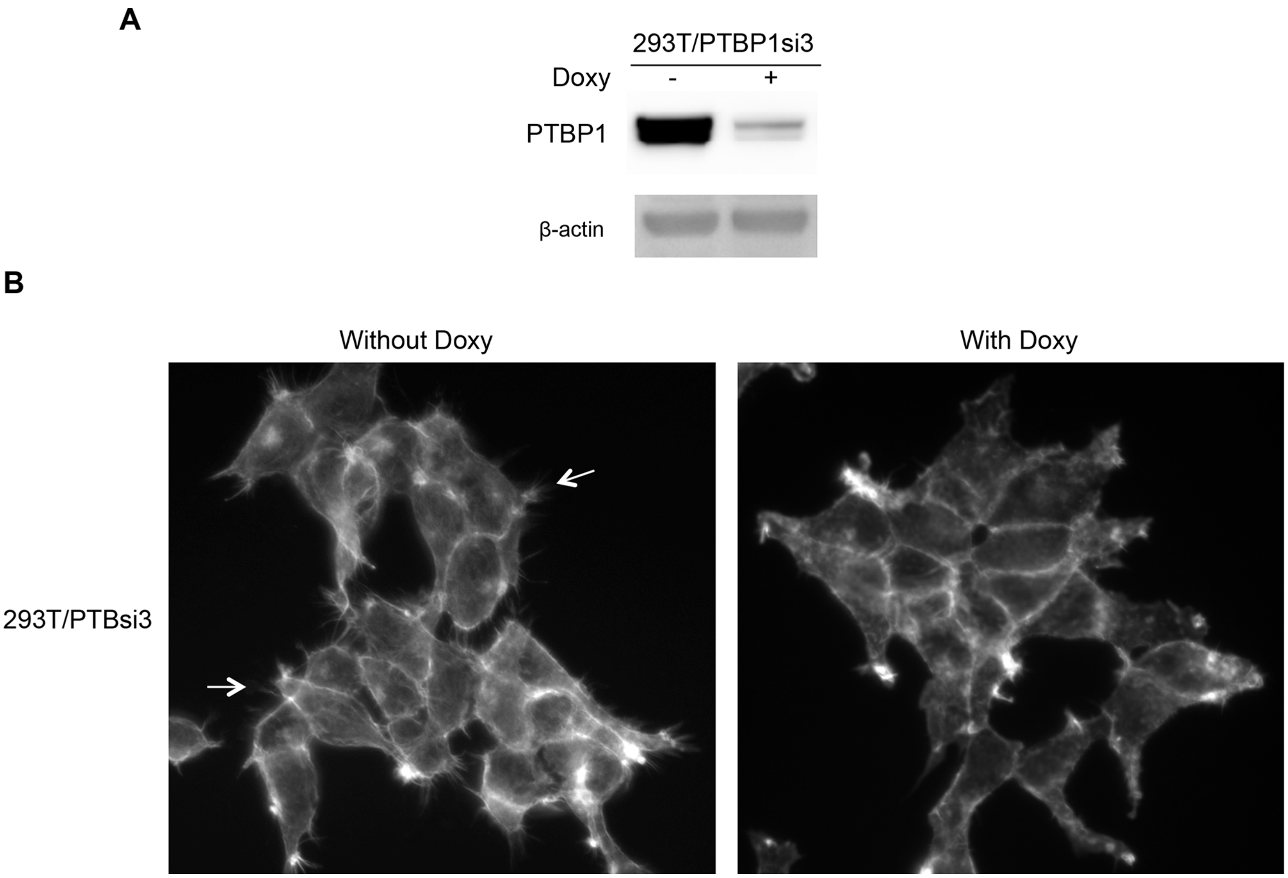

Supplementary Figure S1: Knockdown of PTBP1 inhibits filopodia formation in 293T cells. A. Doxy-induced PTBP1 knockdown in 293T subline cells, 293T/PTBP1si3. **B.** Phalloidin staining. The arrows indicate filopodia. Magnification: 200×.

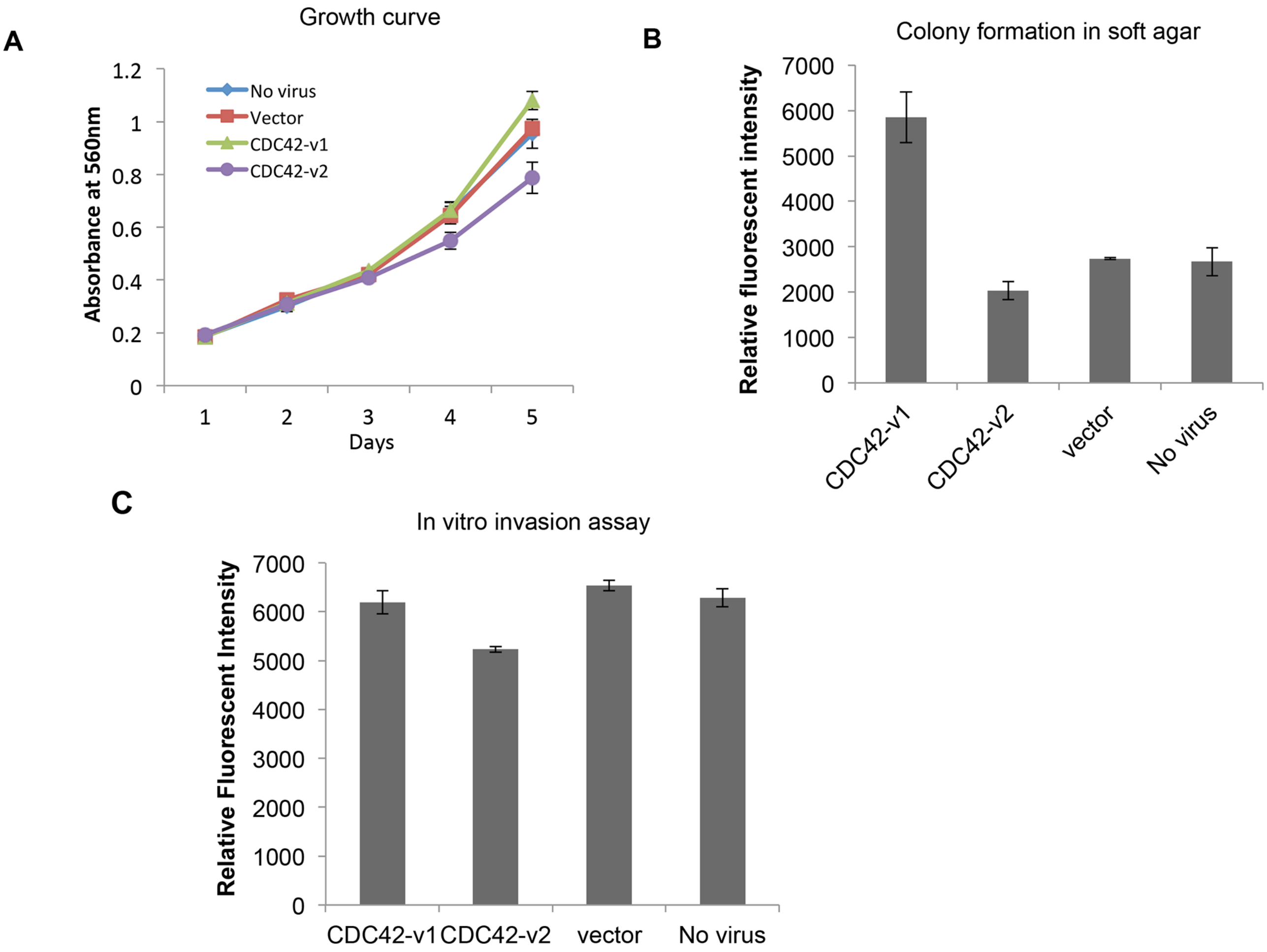

Supplementary Figure S2: Ectopically expressed CDC42-v2 impairs transformation properties of SKOV3 cells. A. Cell growth curve. **B.** Colony formation in soft agar assay. **C.** *In vitro* cell invasion assay. Two separate experiments were performed for each assay and exhibited similar results. Shown in A, B and C are the results of one experiment. Error bar: Standard deviation of quadruplicates.

Exon 6B U/GU-rich region

AAATTAAAAATATACAACCGTTTGT ATAAATGCCTGATGAAGCATCTTTATTCCTGTT

Exon 6A

GTATTAAACAAACAAAAACCCTTC ACAAGCCAGCCTGAGGGTTGTTATTTTCCCTCCGTCTCTTACTTTT

Supplementary Figure S3: Immediate upstream and downstream sequences of polyadenylation sites on exons 6A and 6B of CDC42. The close variant of AATAAA motif is highlighted in red and the U/GU-rich downstream sequence element is highlighted in blue.

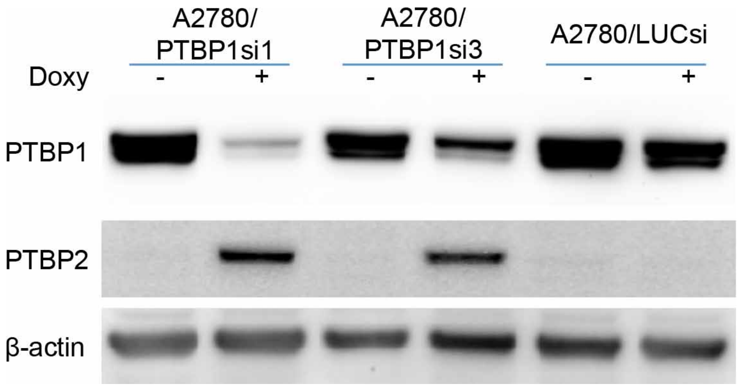

Supplementary Figure S4: PTBP1 knockdown upregulates PTBP2 expression. Shown is the result of western blotting.

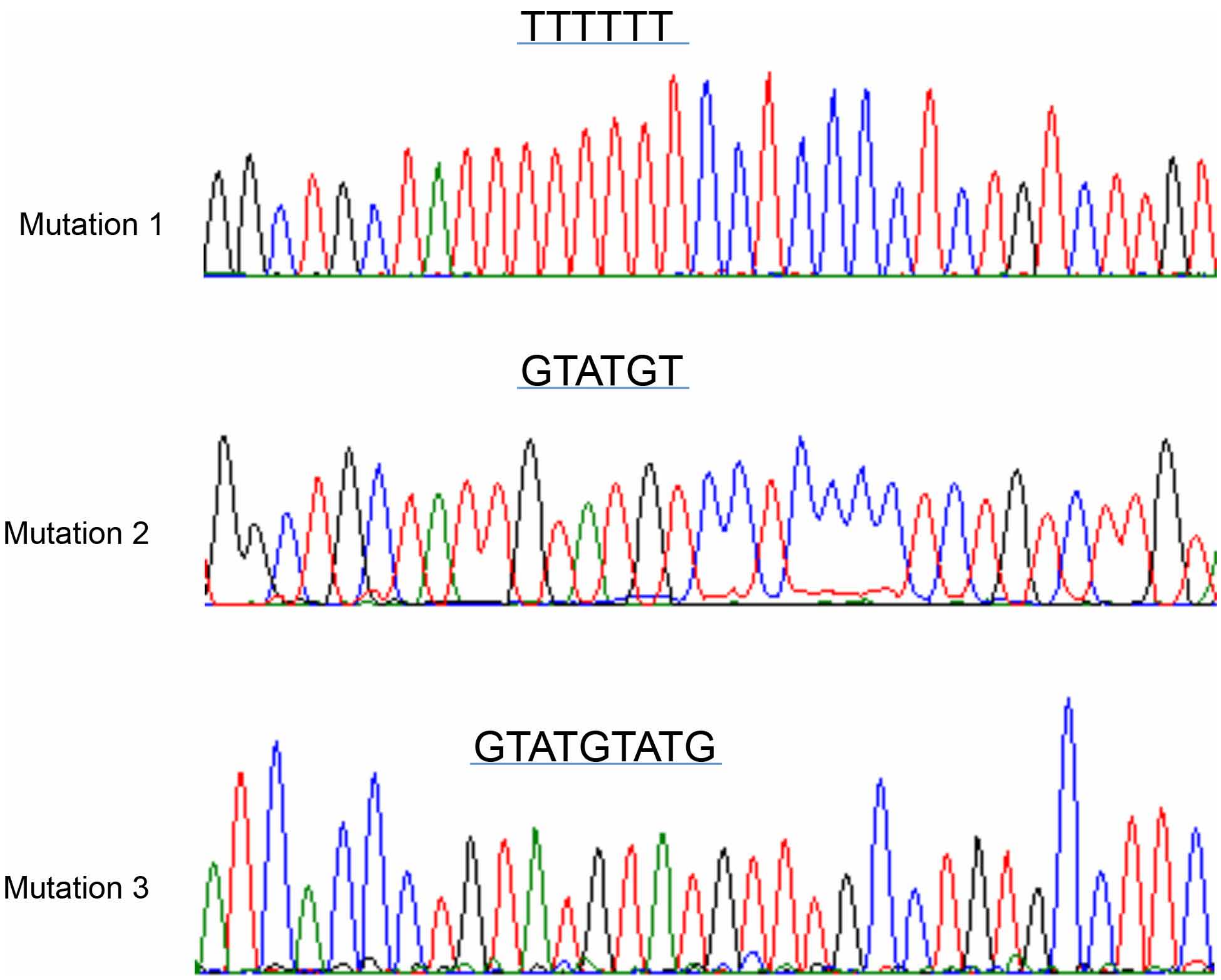

Supplementary Figure S5: DNA sequencing chromatograms showing the mutations introduced into the minigene constructs.
